# Editorial: Microbial community dynamics in agroecosystems: from disease suppression to soil health

**DOI:** 10.3389/fmicb.2026.1860528

**Published:** 2026-05-14

**Authors:** Minchong Shen, Jiangang Li, Jianzhou He, Lin Gao

**Affiliations:** 1 Institute of Tobacco Research, Chinese Academy of Agricultural Sciences, Qingdao, China; 2 Jiangsu Province Engineering Research Center for Agricultural Soil–Water Efficient Utilization, Carbon Sequestration and Emission Reduction, Nanjing, China; 3 College of Agricultural Science and Engineering, Hohai University, Nanjing, China; 4 Department of Biochemistry, Chemistry & Physics, Georgia Southern University, Savannah, GA, United States

**Keywords:** agricultural practices, agroecosystems, disease suppression, microbe-plant interactions, microbial communities, microbial diversity, soil health, sustainable agriculture

The requirement for sustainable agricultural intensification has placed the soil microbiome at the forefront of scientific research. Microbial communities serve as the unseen engines of agroecosystems, driving nutrient cycling, structuring soil, suppressing pathogens, and underpinning plant health. Yet, the very practices that have boosted crop yields—intensive tillage, monoculture, and heavy usage of chemical fertilizer—often disturbs the ecological balance of these communities, creating a paradox where high productivity comes at the cost of long-term soil health and resilience. This Research Topic was conceived to explore the dynamic interaction between agricultural management strategies and soil microbial communities, with the goal of harnessing their potential for disease suppression and ecosystem sustainability.

The collection of articles presented here tackles this challenge from multiple aspects, collectively illustrating that the path to sustainable agriculture lies not in managing soil as a passive substrate, but in cultivating it as a living ecosystem. By integrating high-throughput sequencing, functional gene analysis, and field experimentation, these studies move beyond descriptive accounts of microbial composition to reveal the mechanisms by which management practices can steer the microbiome toward beneficial outcomes.

A central theme emerging from this Research Topic is the power of crop diversification. In a comprehensive review, Zhang Y. et al. elucidated the potential of biological nitrogen fixation (BNF) in rice paddies—a system traditionally reliant on synthetic nitrogen. The authors dissect the paradox that while mineral nitrogen suppresses diazotroph activity, strategic management—such as straw incorporation and the selection of ammonium-tolerant bacterial strains—can sustain BNF within realistic fertilization regimes. This work highlights that enhancing a key ecosystem service is not about replacing synthetic inputs but about optimizing them in concert with biological processes. Complementing this, Liu Y. et al. provided compelling field evidence from a citrus orchard, showing that long-term grass mulching significantly enhances soil nutrients, enzyme activities, and the abundance of beneficial Proteobacteria while reducing fungal pathogens. Crucially, the study links these microbial shifts to improved fruit quality, demonstrating that diversification can create a positive feedback loop where soil health and crop productivity are mutually reinforced.

The principle of diversification is further explored across cropping systems. Liu X. et al. demonstrated that rotating processing tomatoes with maize or seed pumpkin profoundly alters soil microecology. Their work reveals that rotation not only reshapes bacterial and fungal community composition but also enhances network complexity—a topological feature associated with greater functional stability and resilience—contrasting sharply with the simplified, less connected networks found under continuous monoculture, which are more vulnerable to disturbance. Similarly, Gu et al. showed that intercropping tobacco with soybean or maize improves soil fertility and microbial network modularity, fostering a more robust ecosystem. Notably, the study identifies distinct effects: tobacco–soybean intercropping enhanced nitrogen availability via legume symbiosis, while tobacco–maize intercropping enriched functional taxa like Actinobacteria, underscoring that the choice of companion crop can be tailored to specific soil health objectives. In a contrasting case, Wooliver et al. offered a nuanced view from a 4-year field experiment in the United States, finding that while cover cropping and crop rotation did not increase overall microbial diversity, they strongly influenced community composition—reducing fungal plant pathogens and transiently increasing arbuscular mycorrhizal fungi (AMF). This work is a crucial reminder that diversity metrics alone are insufficient; the functional composition of the community is a more sensitive indicator of management impacts. Zhang B. et al. further examined continuous cropping obstacles in Ganoderma leucocontextum cultivation, revealing a shift from bacterial- to fungal-dominated communities over the growth cycle, with Ganoderma itself becoming the dominant genus at maturity alongside decreasing polysaccharides and increasing triterpenoid acids—findings that link microbial succession to both fungal productivity and bioactive compound accumulation.

Beyond diversification, the direct application of soil amendments represents another powerful lever for microbiome management. Zheng et al. investigated the co-application of biochar and Bacillus subtilis in dryland soils, finding a synergistic effect that increased microbial biomass, altered bacterial and fungal community structure, and enhanced enzyme activity. This work exemplifies a “dual-mechanism” approach, where a structural amendment (biochar) and a biological inoculant work in concert to reshape the soil environment and its biota. The theme of synergy is powerfully extended by Chen et al., who explored the combined use of Bacillus spp. with graphene oxide (GO) in the rhizosphere of peach trees suffering from anthracnose. Their results show that the combined treatments were more effective than either agent alone in restoring soil physicochemical properties and microbial community function toward a healthy state. This study opens a new frontier in “nanobio” interventions, suggesting that the unique properties of nanomaterials can be harnessed to potentiate the effects of beneficial microbes.

In the context of disease, Sun et al. identified Ralstonia as the key pathogen in ginger wilt, demonstrating that disease dramatically reshapes the rhizosphere microbiota—reducing beneficial phyla like Acidobacteria and enriching a pathogenic consortium with enhanced stress-tolerance potential. This study underscores the importance of diagnosing the specific microbial dysbiosis associated with a disease to target interventions effectively. The use of microbial inoculants alone is also explored, with Fernández-Pastrana et al. demonstrating that a humic-rich biofertilizer fortified with *Bacillus* or *Pseudomonas* strains not only increased alfalfa yield and quality but also maintained microbial diversity and, intriguingly, lowered soil resistance to β-lactam antibiotics—a novel finding with significant implications for the One Health approach.

While management aims to enhance beneficial microbes, it must also mitigate the negative impacts of agriculture on the climate. Sun et al. addressed this critical intersection by investigating how partial substitution of chemical nitrogen with organic amendments reduces nitrous oxide (N_2_O) emissions in tea plantations. Their structural equation modeling reveals that the substitution alters soil pH and microbial biomass, which in turn modulates the abundance of key denitrification genes (nirS, nirK, and nosZ), ultimately curbing N_2_O release. This work elegantly demonstrates that managing for soil health can simultaneously deliver climate mitigation benefits.

Finally, Wu et al. introduced a novel, temperature-dependent selective soil treatment agent that effectively suppresses tobacco root and stem pathogens while preserving beneficial microbial communities. This agent exemplifies the next generation of “smart” interventions that are ecologically selective, moving away from the broad-spectrum, non-specific action of traditional biocides. The study's demonstration that this selective pressure translates to improved photosynthetic performance in the crop plant closes the loop, linking soil microbial management directly to plant physiological health.

Collectively, these articles advance a transformative vision for agroecosystem management: one where soils are managed not as inert growing media but as dynamic, living systems whose microbial constituents can be deliberately cultivated to support productivity, resilience, and environmental health. The emerging paradigm emphasizes functional outcomes over taxonomic metrics, synergistic interventions over single-agent solutions, and ecological selectivity over broad-spectrum disruption. Future research should prioritize the translation of these mechanistic insights from controlled experiments into practical, scalable management strategies that reconcile the demands of food production with the imperatives of environmental stewardship.

We extend our gratitude to all the authors, reviewers, and editorial staff who contributed to this Research Topic. Their collective efforts provide a rich and compelling foundation for future research aimed at unlocking the full potential of the soil microbiome for a sustainable and food-secure future.

